# Effect of Poly(Alkenoic Acids) Molecular Weight on Microshear Bond Strength of Glass Ionomer Cement to Dentin

**DOI:** 10.1155/ijod/5800346

**Published:** 2026-05-08

**Authors:** Faraz Khodakarami, Mohammad Atai, Zahra Shahidi, Fariba Motevasselian

**Affiliations:** ^1^ School of Dentistry, Tehran University of Medical Sciences, Tehran, Iran, tums.ac.ir; ^2^ Iran Polymer and Petrochemical Institute (IPPI), Tehran, Iran, ippi.ac.ir; ^3^ Department of Restorative Dentistry, School of Dentistry, Tehran University of Medical Sciences, Tehran, Iran, tums.ac.ir

**Keywords:** bond strength, dentin, glass ionomer cement, molecular weights, poly(alkenoic acids)

## Abstract

**Objective:**

To assess the influence of poly(alkenoic acids) (PAA) molecular weight (MW) on the microshear bond strength (µSBS) of conventional glass ionomer cements (GICs) to dentin.

**Materials and Methods:**

Twenty‐four third molars were sectioned mesiodistally to expose flat buccal halves and randomly divided into four main groups. Three PAA solutions were synthesized with different MWs: (i) high molecular weight (HMW: Mw = 46,300 Da), (ii) low molecular weight (LMW: Mw = 15,700 Da), and (iii) mixed molecular weight (MixMW: 1:1 mixture of HMW and LMW polymers). The PAA solutions were hand‐mixed with a commercial GIC restorative powder (Fuji II, GC Corp., Tokyo, Japan) at a powder‐to‐liquid ratio of 1.5:1 (wt/wt). Each tooth specimen received microcylinders at the mesiobuccal and distobuccal line angles from the four tested materials, including the control group and the experimental groups. µSBS was tested after subjecting the specimens to thermocycling (5000 cycles; 5–55°C) using a universal testing machine at a crosshead speed of 0.5 mm/min. Data were analyzed using a one‐way ANOVA test (*p*  < 0.05). Failure modes were assessed using a stereomicroscope at 40× magnification.

**Result:**

There was a significant difference between the groups. There was a significant difference between the groups. The mixture of PAA containing both HMW and LMW exhibited higher µSBS values compared with both the HMW group and the control group. Mixed failures were the most common, while adhesive failures occurred least frequently among the experimental groups.

**Conclusion:**

The selected mixed ionomers of different MWs had a statistically significant effect on the µSBS of GIC to dentin.

## 1. Introduction

Glass‐ionomers (GIs) are self‐adhesive restorative dental materials that provide chemical adhesion to enamel and dentin [1]. They are used clinically as cavity liners or bases [2], luting cements, and for the restoration of natural teeth [3, 4]. Although GIs do not possess the same physical and mechanical strength as resin‐based composites, they offer other advantages that make them valuable in restorative dentistry and essential for clinical practice [1].

These advantages include resistance to microleakage, good marginal integrity, a coefficient of thermal expansion similar to that of tooth structure, biocompatibility, fluoride release and rechargeability, and reduced shrinkage upon setting, with no free monomer being released [1, 5].

GICs are formed by the reaction between an ion‐leachable glass and poly(alkenoic acids) (PAA) in an aqueous solution [3]. The powder consists of basic glass particles, typically calcium or strontium alumino‐fluoro‐silicate, with added phosphate and some sodium [6]. These particles react with the polymeric solution to form salts that crosslink the polymer chains, leading to hardening of the material [1, 6–9]. The PAA are either homopolymers or copolymers of monomers such as acrylic acid, maleic acid, and itaconic acid [4, 6].

The adhesion of GIs to the tooth surface is a crucial clinical property, as it helps retain the cement within the tooth and reduces microleakage [6]. Adhesion occurs in multiple stages: initially, the fresh cement paste wets the tooth surface, followed by the formation of ionic bonds between cations in the tooth and anionic functional groups in the cement [6, 10].

Several strategies have been employed to enhance the properties of GICs, particularly their mechanical performance [6, 11]. These include modifications to the GI powder composition [7, 12, 13], incorporation of nanoparticles [8, 14, 15], and the use of fibers [16, 17]. Mechanical properties can also be improved by optimizing the polymeric matrix, for example, by adjusting the molecular weight (MW) of PAA, its distribution, or its concentration [2, 3, 9, 18].

Increasing the MW of PAA is expected to influence several clinically relevant properties, including viscosity, handling characteristics, setting rate, and working time [2, 3, 9, 18]. Therefore, an optimal PAA concentration should be selected for each Mw to maintain favorable mechanical properties [2, 3, 9, 18]. The polymer architecture may be linear, branched, or a combination of both [19].

Several studies were performed to investigate how polymer MW and its ratio affect GICs mechanical properties [2, 3, 9, 18]. However, to the best of our knowledge, there is limited information available on the polymeric acid MW of GICs on the bond strength to dentin.

Therefore, experimental solutions of PAAs with different MWs were prepared to evaluate this feature on dentin bond strength. It was null hypothesized that the MW of PPA has no effect on bond strength to dentin.

## 2. Materials and Methods

### 2.1. Ethical Considerations

This study was approved by the Ethics Committee of the School of Dentistry, Tehran University of Medical Sciences (IR.TUMS.BLC.1402.019) and conducted in accordance with the Declaration of Helsinki. Extracted human third molars used in this study were removed for treatment reasons unrelated to this research and were anonymized prior to use.

### 2.2. Sample Size Calculation

The required sample size was estimated using the two‐sample *t*‐test module in PASS 11 software. Using a significance level of 0.05, a power of 80% (*β* = 0.20), and a standard deviation of 3.2 MPa reported by El‐Wakeel et al. [20], the analysis indicated that a minimum of 12 specimens per group were required.

### 2.3. Ionomers Preparation With High and Low MW

The powder component of a commercially available hand‐mixed GI restorative (Fuji II; GC Corp, Tokyo, Japan, shade A3) was used for all specimens in this study. PAA with an acrylic acid/itaconic acid mass ratio of 70/30 were synthesized following a previously reported procedure [4]. Briefly, the polymerization conditions—including initiator, solvent, and temperature—were as described in the reference to ensure reproducibility. The weight‐average molecular weight (*Mw*), number‐average molecular weight (*Mn*), and polydispersity index (PDI, *Mw/Mn*) of the two experimental PAAs were determined using gel permeation chromatography [4].

Ionomer solutions were prepared consisting of 40 wt% polymer, 5 wt% tartaric acid (Aldrich, Germany), and 55 wt% deionized water. Three experimental groups were formulated: (i) high molecular weight (HMW) group, containing polymer with Mw = 46,300 Da, Mn = 20,900 Da, PDI = 2.2; (ii) low molecular weight (LMW) group, containing polymer with Mw = 15,700 Da, Mn = 8,730 Da, PDI = 1.36; and (iii) mixed molecular weight (MixMW) group, containing a 1:1 mixture of the HMW and LMW polymers. Additionally, the commercially available Fuji II GI cement (GC Corp, Tokyo, Japan, shade A3) was used as the control group.

### 2.4. GI Restorative Preparation

A powder‐to‐liquid (P/L) ratio of 2.7:1 (wt/wt) was initially used for each GIC group according to the manufacturer’s instructions. However, the mixtures were too viscous, and rapid setting limited placement of the GIC into the silicone tubes for the microshear test. To obtain a workable consistency, several P/L ratios were tested, and an optimal ratio of 1.5:1 was selected for all groups. For preparation, powders and liquids were weighed using a digital balance with 0.1 mg accuracy (Kern, Germany) and mixed in a silicone jar. The powder and liquid were hand‐mixed thoroughly for 15 s.

### 2.5. Specimen Preparation

A total of 24 sound human third molars, extracted within the last 3 months, were collected. The teeth were cleaned and stored in 1% chloramine T solution for 1 week, then kept in distilled water at 4°C until use. All teeth were examined under × 20 magnification to exclude those with structural defects. The teeth were mounted in self‐polymerizing acrylic resin (Acropars, Marlic Co., Tehran, Iran) up to 2 mm below the cementoenamel junction (CEJ).

A mesiodistal section was made on the buccal surface using a diamond disc (Mecatom T201, Pressi, France) to expose the buccal one‐third of the dentin. The buccal surfaces were then wet‐ground with 600‐grit silicon carbide paper to create smooth dentin surfaces. The dentin was rinsed with water and gently blot‐dried. Mesiobuccal and distobuccal line angles, located 2 mm above the CEJ, were selected for GIC application. The specimens were randomly divided into four groups (*n* = 12) according to the MWs of the ionomer solutions.

The mixed cements were condensed into silicone tubes (1.5 mm height × 1 mm diameter). A transparent strip was placed over the GIC surface and gently pressed to ensure complete adaptation of the cement to the dentin. Pressure was maintained until the initial setting of the GIC. The exposed surface of the cement was then covered with petrolatum to prevent moisture loss or absorption. Specimens were stored in an incubator at 37°C and 100% humidity for 24 h. After this period, the silicone tubes were carefully removed using a sharp blade. The GIC cylinders were examined under a stereomicroscope, and any specimens with cracks or voids at the interface or within the bulk material were excluded. The remaining specimens were subjected to 5000 thermal cycles between 5°C and 55°C water baths, with a dwell time of 20 s per bath, simulating ~6 months of clinical service [21].

### 2.6. Microshear Bond Strength (μSBS) Test

μSBS testing was performed using a universal testing machine (STM‐20, Santam, Iran) equipped with a metal jig, a 6 kg load cell, and a crosshead speed of 0.5 mm/min. A thin wire loop (0.14 mm diameter) was placed around each cylindrical specimen as close as possible to its base. The μSBS (in MPa) was calculated by dividing the peak load at failure (N) by the bonded surface area (mm^2^) of the specimen.

After debonding, the fractured surfaces of each specimen were examined at 40× magnification under a stereomicroscope (Olympus Z61, Japan). Failure modes were classified as cohesive (fracture within the GIC cylinder), adhesive (failure at the cement–dentin interface), or mixed (combination of cohesive and adhesive failure).

## 3. Statistical Analysis

The data were processed and analyzed using the Statistical Package for the Social Sciences software, version 25.0 (SPSS Inc., Chicago, Illinois, USA) and One‐Way ANOVA. Statistical significance was set at *p*‐value less than 0.05. Data normality and homogeneity of variance were verified using the one‐sample Kolmogorov–Smirnov test. The statistical differences were then examined by one‐way ANOVA and Bonferroni post hoc test at a significant level of *p*  < 0.05.

## 4. Results

**Figure 1:**
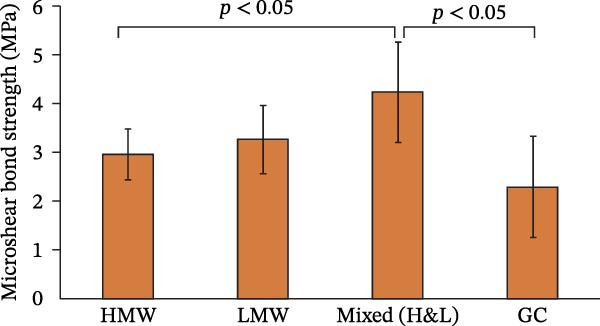
Microshear bond strength (µSBS) in MPa.

The μSBS values are presented in Table 1 and Figure 1.

**Table 1:** Microshear bond strength (µSBS) values (MPa) of the control and study groups presented as mean and standard deviation.

Groups	Microshear bond strength (MPa) mean ± SD^∗^
GC	2.29 ± 1.04^a^
HMW	2.96 ± 0.52^a^
LMW	3.26 ± 0.70^ab^
Mixed (H&L)	4.23 ± 1.03^b^

Abbreviations: GC, control group (Fuji ii; GC); HMW, high molecular weight of poly(alkenoic acids); LMW, low molecular weight of poly(alkenoic acids); Mixed (H&L), 1:1 mixture of high and low molecular weight of poly(alkenoic acids).

^∗^Groups sharing the same superscript letter are not significantly different (*p*  > 0.05).

One‐way ANOVA revealed a statistically significant difference between the mixed (H&L) group and both the control (GC) and HMW groups (*p*  < 0.05). No statistically significant differences were observed among the remaining groups (*p*  > 0.05).

**Figure 2:**
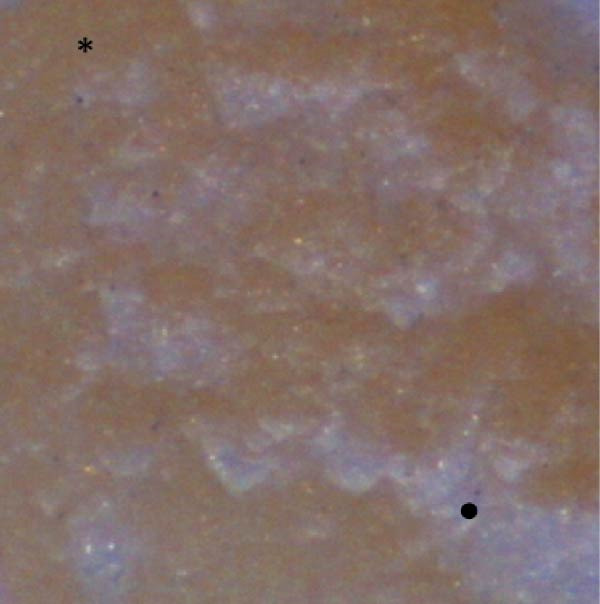
Representative image of a mixed failure mode observed in a specimen.  ^∗^ indicates the dentin region and • indicates the glass ionomer cement region on the specimen surface.

Failure modes for specimens are shown in Table 2. Figure 2 illustrates a representative mixed failure mode observed in a specimen. Mixed and adhesive failures had the highest and lowest frequency in the experimental groups, respectively. In the control group, Fuji ii GC, the predominant failure mode was adhesive and cohesive failure exhibited the lowest failure mode.

**Table 2:** Failure mode distribution in the control and study groups (%).

Groups	Cohesive (%)	Adhesive (%)	Mixed (%)
GC	25	41.6	33.3
HMW	33.3	16.6	50
LMW	33.3	8.3	58.3
Mixed (H&L)	16.6	8.3	75

Abbreviations: GC, control group (Fuji ii; GC); HMW, poly(alkenoic acids) with high molecular weight; LMW, poly(alkenoic acids) with low molecular weight; Mixed (H&L), mixture of high and low molecular weight of poly(alkenoic acids).

## 5. Discussion

This study investigated the effect of PAA MW on the µSBS of glass ionomer cement (GIC) to dentin. The results demonstrated that the use of a mixture of high and low MW PAAs significantly increased µSBS compared with both the HMW PAA group and the control group (Fuji II GIC). Consequently, the null hypothesis was rejected. No statistically significant differences were observed among the remaining groups. The bond strength values obtained in the present study fall within the reported range for conventional GICs (1.32–4.1 MPa), indicating consistency with previous findings [22].

The bond strength of GIC to dentin is governed by both interfacial adhesion mechanisms and the cohesive properties of the cement. Interfacial adhesion is primarily mediated by chemical interactions between the carboxylate groups of PAA and calcium ions in dentinal hydroxyapatite, resulting in the formation of an ion‐exchange layer at the interface, with a limited contribution from micromechanical interlocking. In parallel, the cohesive strength of the cement is determined by the polymer network formed during the acid–base reaction, which is influenced by polymer MW, crosslink density, and polymer–glass interactions [6, 23, 24].

The MW of PAA plays a critical role in modulating these mechanisms through its influence on viscosity, chain mobility, polymer entanglement, and network formation [2, 3, 9, 18, 19]. Lower MW polyacids exhibit reduced viscosity and greater chain mobility [2, 3, 9, 18], facilitating improved wetting of the dentin surface and closer interfacial contact [6]. However, shorter polymer chains limit chain entanglement, which may reduce the cohesive strength of the set cement [2, 3, 9, 18]. In contrast, higher MW polyacids produce more viscous solutions, which can restrict polymer mobility and potentially compromise interfacial adaptation, while simultaneously increasing chain entanglement and cohesive strength within the cement matrix [2, 3, 6, 9, 18].

The µSBS test induces a complex stress state involving both tensile and shear components within the cement and at the cement–dentin interface [25]. It has been reported that bond strength values obtained in such experimental tests often reflect the tensile strength of the cement during load application rather than true interfacial adhesive strength [6]. Nevertheless, despite its complex stress distribution, the µSBS test is still commonly employed to evaluate dentin bonding owing to its simplicity, reproducibility, and suitability for comparative analyses [26]. In this context, several studies have shown that failures in GIC during shear bond testing are frequently cohesive, occurring within the cement bulk rather than at the interface [6, 27]. Therefore, failure mode analysis provides complementary qualitative information that aids in interpreting whether changes in µSBS are predominantly associated with interfacial adaptation, cohesive properties of the cement, or a balance of both mechanisms [6, 23, 27].

Based on previous studies, PAA MW can influence working and setting times, viscosity, and the formation of the polymer network, including ionic crosslinking with glass particles [2, 3, 9, 18, 19]. Wilson et al. [18] reported that increasing the MW of PAA in GIC liquids increases viscosity, reduces working time, and accelerates gel formation, depending on polymer concentration Although chemical properties were not measured in the present study, the observed differences in microshear bond strength and failure modes among the low, high, and mixed MW groups—at the modified P/L ratio used to achieve workable consistency—may reflect beneficial effects of polymer chain length on both cohesive strength and interfacial bonding.

Previous work by Wilson et al. [18] highlighted the complex influence of PAA MW on the cohesive behavior of GICs. While higher MW polymers increase network strength through enhanced entanglement, lower MW polymers tend to generate smaller flaws and maintain fracture resistance through ionic crosslinking and effective glass–polymer interactions [18]. This interplay may explain the superior µSBS observed in the MixMW group, where combining short and long polymer chains likely produces a more uniform polymer network, improves stress distribution, and reduces the likelihood of catastrophic crack propagation under load.

The lower µSBS of the control group compared with the mixed group may be related to the lower powder‐to‐liquid (P/L) ratio used, relative to the manufacturer’s recommended ratio, to standardize consistency across groups. Since cement strength also depends on the proportion of filler to matrix and the adhesion between filler particles and the matrix [3], this difference may partially explain the observed result. It is important to note that the exact composition of PAA in the GC liquid is not publicly available, so this finding cannot be fully explained.

Failure mode analysis in the present study supports this interpretation. Although adhesive failures were most frequent in the control group, the MixMW group exhibited a predominance of mixed failures. This pattern suggests a more balanced contribution of cohesive integrity and interfacial bonding in the mixed group, rather than failure being dominated by a single weak zone. The lower incidence of adhesive failures observed mixed groups further indicates relatively effective interfacial adaptation in this formulation. While failure mode assessment does not provide a quantitative measure of bond strength, these qualitative observations are consistent with the enhanced µSBS recorded for the MixMW group and reinforce the concept that optimizing both cohesive and interfacial properties is essential for improving GIC performance.

Although no previous studies have directly evaluated the effect of PAA MW on the dentin bond strength of GICs, several investigations have emphasized the importance of balancing polymer MW, concentration, and architecture to optimize mechanical performance [2, 3, 9, 18, 19]. In agreement with these findings, the present results suggest that combining HMW and LMW PAAs can achieve a favorable balance between cohesive strength and interfacial adaptation, leading to improved bonding performance. This indicates that principles governing the bulk mechanical behavior of GICs may also extend to their interfacial bonding characteristics.

The present study evaluated a limited range of linear PAAs, which may have restricted the magnitude of observable differences among groups. Future studies should explore a broader range of PAA MWs, blend ratios, and polymer architectures, including branched and polydisperse systems, to further elucidate the mechanisms governing both interfacial adhesion and cohesive strength. Such investigations may contribute to the optimization of GIC formulations with enhanced clinical performance.

## 6. Conclusion

Our findings suggest that a mixture of HMW and LMW PAA provides higher dentin bond strength and increases the proportion of mixed failures. These results may inform the design of GIC formulations with mixed polymer MWs to enhance bonding performance.

## Funding

The authors have nothing to report.

## Conflicts of Interest

The authors declare no conflicts of interest.

## Data Availability

The data that support the findings of this study are available upon request from the corresponding author. The data are not publicly available due to privacy or ethical restrictions.
